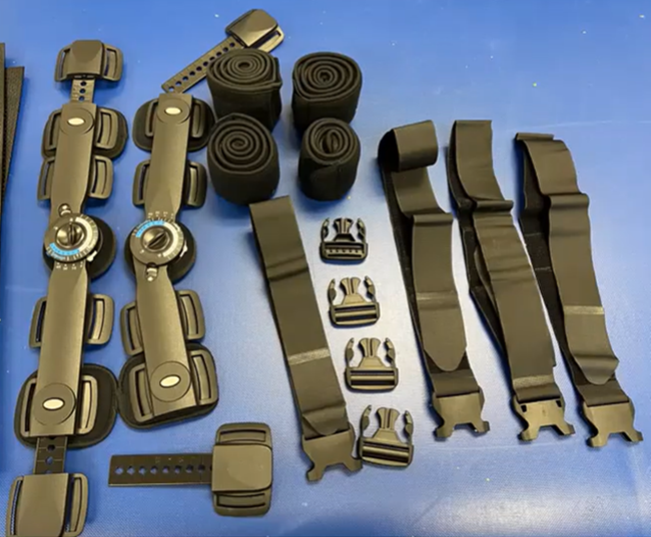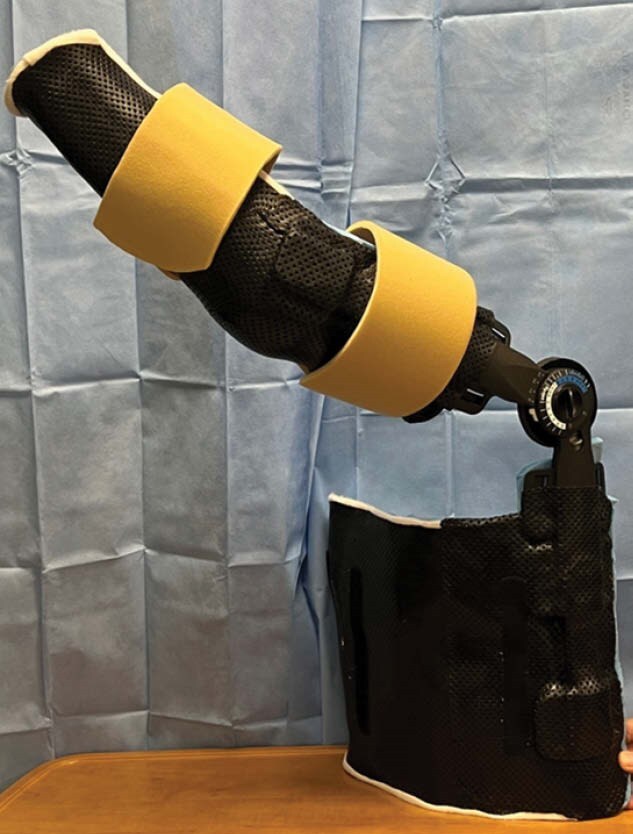# 1007 Utilization of Alternate Hinge for Static Progressive Shoulder Orthosis in Adults: A Case Report

**DOI:** 10.1093/jbcr/iraf019.538

**Published:** 2025-04-01

**Authors:** Andria Martinez, Claudia Islas, Renee Warthman, Karen Richey, Kevin Foster

**Affiliations:** Diane & Bruce Halle Arizona Burn Center; Diane & Bruce Halle Arizona Burn Center; Diane & Bruce Halle Arizona Burn Center; Diane & Bruce Halle Arizona Burn Center; Diane & Bruce Halle Arizona Burn Center

## Abstract

**Introduction:**

Rehabilitative management of patients with large total body surface area (TBSA) and burns to the axillae present unique challenges to maintain range of motion (ROM). Although prefabricated devices are available, they are limited in number and cater to orthopedic populations. Burn rehabilitation therapists often fabricate shoulder positioning devices out of foam, PVC pipe, etc. However, these materials also pose challenges with respect to infection control, longevity, size, and can limit mobility out of bed. Utilization of a locking hinge allows the patient to be positioned from a cutaneokinematic perspective and place their tissues in an elongated position for an extended period of time, which ultimately combats adaptive shortening. The purpose of this case study is to propose the use of a locking hinge from a knee brace and subsequently utilize it when fabricating a custom shoulder positioning device in adult patients.

**Methods:**

This study utilized a single case report. Data collected was the cost of the hinge, cost of thermoplastic materials, number of times the device was used from fabrication to discharge, total number of device applications, average number of minutes the device was worn, and total number of minutes used.

**Results:**

The was a 31-year-old male with 48% TBSA flame burn. The cost of a single hinge used may be $28.86 - $42.67, depending on the size of the hinge needed. The cost of the thermoplast used may be $36.02 - $54.09 pending the size of material needed. The total approximate cost of these materials is $75.81. Once the orthotic was fabricated, it was used over a span of 85 days and applied for a total of 26 times. The device was worn for an average of 45 minutes. This allowed the patient’s extremity to be positioned in a safe and controlled manner for 1,170 minutes, of which was monitored by nursing, family and the therapy teams.

**Conclusions:**

In this case, use of an alternate locking hinge to fabricate a custom shoulder flexion orthosis may decrease the overall cost of treatment, improve device longevity, limit the need of total positing devices for the axilla and improve the length of time at which tissue can be placed in an elongated position. This device also allowed for a static progressive stretch through use of a hinge and more precise positioning based on the patient’s cutaneous involvement to provide a more custom approach.

**Applicability of Research to Practice:**

Although the positioning needs of a burned axilla vary over time, utilization of a locking hinge in a custom orthosis may decrease the overall cost and number of orthoses required to maintain and improve tissue length.

**Funding for the Study:**

N/A